# Correlation analysis of the expression levels of serum endothelial cell adhesion molecule-1 and sirtuin 1 protein in acute respiratory distress syndrome

**DOI:** 10.5937/jomb0-61088

**Published:** 2026-06-16

**Authors:** Can Peng, Zhigang Sun, Yanyan Liu, Yougui Liu, Hao Zhao

**Affiliations:** 1 Zhejiang Chinese Medical University, Graduate School, Department of Infectious Diseases, Jiashan County, Zhejiang Province, China; 2 Peking University First Hospital, Department of Laboratory Medicine, Beijing City, China; 3 Hangzhou Traditional Chinese Medicine Hospital Affiliated to Zhejiang Chinese Medical University, Department of Paediatrics, Hangzhou City, China; 4 The First Affiliated Hospital of Zhejiang Chinese Medical University (Zhejiang Provincial Hospital of Chinese Medicine), Department of Emergency, Hangzhou City, China

**Keywords:** sepsis-associated acute respiratory distress syndrome, silent information regulatory factor 2-related enzyme 1, endothelial cell-specific molecule-1, treatment outcome, associated fibroblast growth factor-21, akutni respiratorni distres sindrom povezan sa sepsom, enzim povezan sa tihim regulatorom informacija 2, molekul specifičan za endotelne ćelije 1, faktor rasta fibroblasta-21, ishod lečenja

## Abstract

**Background:**

To investigate the relationships among the expression levels of serum silencing information regulator 2-related enzyme 1 (SIRT1), endothelial cell-specific molecule-1 (ESM-1), and fibroblast growth factor-21 (FGF21) and treatment results in patients with acute respiratory distress syndrome (ARDS) associated with sepsis.

**Methods:**

A total of 140 patients with sepsis-related ARDS were selected and divided into a good-outcome group (96 patients) and a poor-outcome group (44 patients) based on treatment outcome. The levels of serum SIRT1, ESM-1, and FGF21 were compared between the two groups; the correlations between serum SIRT1, ESM-1, and FGF21 and disease severity and treatment outcome were analysed; and the predictive value of serum SIRT1, ESM-1, and FGF21 for treatment outcomes was evaluated.

**Results:**

Compared with those in the group with favourable outcomes, the serum SIRT1 level was lower, while the ESM-1 and FGF21 levels were significantly higher in the poor outcome group than in the good outcome group (P&lt;0.05). Serum SIRT1 levels decreased steadily in patients with mild, moderate, and severe illness, whereas ESM-1 and FGF21 levels increased consistently (P&lt;0.05). ESM-1 and FGF21 showed a positive association with illness severity (P&lt;0.05), whereas serum SIRT1 was negatively correlated (P&lt;0.05) with disease severity according to Spearman correlation analysis. Partial correlation analysis indicated that serum SIRT1, ESM-1, and FGF21 levels were significantly related to treatment outcomes in patients with sepsis-related ARDS (P&lt;0.05). The levels of serum SIRT1, ESM-1, and FGF21 and treatment outcomes were strongly linked (P&lt;0.05) in patients with sepsis-related ARDS. The areas under the curve (AUCs) for predicting treatment course in patients with ARDS associated with sepsis were 0.742 for SIRT1, 0.838 for ESM-1, and 0.796 for FGF21. The sensitivities were 77.27% and 70.45%, and the specificities were 64.58%, 81.25%, and 87.50%, respectively. For patients with sepsis-related ARDS, the combined AUC of the three markers for treatment outcome was 0.939, with a sensitivity of 88.64% and a specificity of 83.33%, significantly surpassing the individual predictive values of the three markers alone (P&lt;0.05).

**Conclusions:**

The levels of serum SIRT1, ESM-1, and FGF21 in patients with sepsis-related ARDS are strongly associated with both treatment efficacy and illness severity, can independently predict treatment outcomes, and have greater combined predictive value.

## Abstract (sr)

**Uvod:**

Cilj je bio da se ispita veza između nivoa ekspresije seruma SIRT1 (enzim povezan sa regulatorom informacija 2), ESM-1 (molekul specifičan za endotelne celije) i FGF21 (faktor rasta fibroblasta-21) i ishoda lečenja kod pacijenata sa sindromom akutnog respiratornog distresa (ARDS) povezanim sa sepsom.

**Metode:**

U studiju je uključeno 140 pacijenata sa ARDS izazvanim sepsom, koji su podeljeni u grupu sa povoljnim ishodom (96 pacijenata) i grupu sa nepovoljnim ishodom (44 pacijenta) na osnovu rezultata lečenja. Nivoi seruma SIRT1, ESM-1 i FGF21 upoređeni su između dve grupe. Analizirane su korelacije između ovih biomarkera i težine bolesti i ishoda lečenja, kao i prediktivna vrednost pojedinačnih markera za ishod lečenja.

**Rezultati:**

U grupi sa nepovoljnim ishodom, nivo seruma SIRT1 bio je značajno niži, dok su nivoi ESM-1 i FGF21 bili znatno viši u poređenju sa grupom sa povoljnim ishodom (P&lt; 0,05). Nivoi SIRT1 postepeno su opadali kako je bolest bila teža, dok su nivoi ESM-1 i FGF21 postepeno rasli (P&lt; 0,05). Spirmanova korelaciona analiza pokazala je da su ESM-1 i FGF21 pozitivno, a SIRT1 negativno povezan sa težinom bolesti (P&lt; 0,05). Parcijalna analiza korelacije pokazala je da su nivoi SIRT1, ESM-1 i FGF21 značajno povezani sa ishodom lečenja kod pacijenata sa sepsom povezanom sa ARDS-om (P&lt; 0,05). Povrsine ispod krive (AUC) za predviđanje toka lečenja kod pojedinačnih markera iznosile su 0,742, 0,838 i 0,796, sa osetljivostima od 77,27% i 70,45% , i specifičnostima od 64,58% , 81,25% i 87,50% , redom. Kombinovana AUC vrednost sva tri markera za predviđanje ishoda lečenja bila je 0,939, sa osetljivosću od 88,64% i specifičnošću od 83,33% , sto je značajno vise od prediktivne vrednosti pojedinačnih markera (P&lt; 0,05).

**Zaključak:**

Nivoi seruma SIRT1, ESM-1 i FGF21 kod pacijenata sa ARDS povezanim sa sepsom snažno su povezani sa težinom bolesti i efikasnošću lečenja, mogu samostalno predvideti ishod lečenja i imaju veću kombinovanu prediktivnu vrednost.

## Introduction

According to relevant statistical data [1][2], sepsis-related acute respiratory distress syndrome (ARDS) significantly increases the risk of poor prognosis in sepsis patients. It can cause 40% to 60% of sepsis patients to die. Early prediction of the treatment outcome of sepsis-related ARDS is crucial for adjusting the treatment plan, enhancing the therapeutic effect, and reducing the risk of death. Silent information regulator 2-related enzyme 1 (SIRT1) is involved in pathological processes such as oxidative stress, apoptosis, and the inflammatory response, and is closely associated with sepsis [3]. The soluble circulating proteoglycan known as endothelial cell-specific molecule-1 (ESM-1) has a wide range of biological functions. It can play a regulatory role in vascular endothelial injury and inflammatory responses via multiple signalling pathways and is closely associated with inflammatory diseases [4]. Fibroblast growth factor-21 (FGF21) is significantly elevated in systemic inflammatory conditions and exacerbates inflammation [5].

Sepsis-associated acute respiratory distress syndrome (ARDS) is a condition characterised by a high mortality rate and a complex course in critical care. Its development involves multiple pathological pathways, including inflammatory storms, endothelial injury, microcirculatory disorders, and metabolic imbalances [6][7][8]. Achieving early risk stratification and evaluating treatment efficacy using repeatable, easily accessible serum biomarkers remains a key challenge in clinical management. Silent information regulator 1 (SIRT1) regulates NF-kB-mediated inflammatory responses, oxidative stress, and mitochondrial homeostasis [9]. Endothelial cell-specific molecule-1 (ESM-1) reflects changes in endothelial activation and permeability, indicating impairment of the pulmonary microvascular barrier and inflammatory exudation. Fibroblast growth factor 21 (FGF21) is a stress-related metabolic hormone with potential roles in metabolic reprogramming, anti-inflammatory effects, and organ protection [10]. Previous studies [11][12][13] have suggested that these indicators are associated with the severity of sepsis, organ dysfunction, and the prognosis of ARDS. However, evidence concerning the combined change characteristics of these three factors in populations with sepsis-related ARDS, their correlation, and their prognostic value for treatment outcomes (such as recovery of organ function and mortality) remains insufficient [14][15][16].

This study aimed to detect the baseline levels and dynamic changes in serum SIRT1, ESM-1 and FGF21 levels in patients, systematically evaluate their correlation with clinical efficacy outcomes and independent predictive ability, and explore the discriminative efficacy of multi-index combined modelling, with the hope of providing a translational biological basis for precise monitoring of disease progression, optimising treatment strategies and improving patient prognosis.

## Materials and methods

### General information

The study included 140 patients with sepsis-related acute respiratory distress syndrome (ARDS) between January 2023 and January 2025.

The inclusion criteria were as follows: (1) aged 18 years or above; (2) met the diagnostic criteria for sepsis; (3) met the diagnostic criteria for ARDS; (4) did not receive relevant treatment before admission; and (5) signed the informed consent form after being made aware of the research procedure.

The exclusion criteria were as follows: (1) patients with concurrent hematological diseases; (2) those accompanied by other acute or chronic inflammatory reactive diseases; (3) those with concurrent immunosuppressive or hyperimmune diseases; (4) those with a history of lung diseases such as asthma, bronchiectasis, and lung tumors; (5) people who suffer from serious heart and brain conditions; (6) those with malignant tumors; (7) those with a history of lung trauma and surgery; (8) those with other organ damage related to sepsis; and (9) patients who were not admitted to the hospital for the first time due to sepsis, those with ARDS caused by other reasons, and pregnant or lactating female patients.

This study was approved by the hospital Ethics Committee (Ethics Review Approval Number: GYZL-ZN-2023-055).

### Data collection

Clinical data, including sex, age, basic medical history, infection site, disease severity, mechanical ventilation duration, and intensive care unit stay duration, were collected for each patient. Disease severity was classified according to the oxygenation index (OI) at admission. Arterial partial pressure of oxygen (PaO_2_) was measured using an ABL90 FLEX arterial blood gas analyser (Radiometer, Denmark), and OI was calculated as PaO_2_ divided by inhaled oxygen concentration. An OI >200 mmHg was considered mild, 100-200 mmHg moderate, and <100 mmHg severe, according to standard ARDS classification.

### Laboratory biochemical testing equipment and reagents

The experimental method used in this study to detect the protein expression levels of SIRT1, ESM-1 and FGF21 in serum was enzyme-linked immunosorbent assay (ELISA).

Human SIRT1 ELISA Kit: Manufacturer: Cloud-clone Corp (Cloud Clone). Country: The United States. Item number: SEA896Hu. Detection principle: Double-antibody sandwich method. The detection range was 0.156-10 ng/mL. Sensitivity: <0.056 ng/mL.

Human ESM-1 (Endocan) ELISA Detection Kit. Manufacturer: RayBiotech, Inc. (RayBio). Country: The United States. Item No.: ELH-Endocan. Detection principle: Double-antibody sandwich method. The detection range was 16-5000 pg/mL. Sensitivity: <8 pg/mL.

Human FGF21 Quantikine® ELISA Kit. Manufacturer: R&D Systems, Inc. (R&D Systems Company, now a brand under Bio-Techne). Country: The United States. Item No.: DF2100 detection principle: Double-antibody sandwich method. The detection range was 3.9-250 pg/mL. Sensitivity: <1.0 pg/mL.

### Laboratory biochemical testing methods

All reagents were equilibrated to room temperature (approximately 25°C) before use, then reconstituted, diluted, or prepared as instructed for each ELISA kit. Repeated freezing and thawing of the kit components were avoided. The serum was collected, centrifuged, aliquoted and frozen in strict accordance with the methods above. Before testing, the samples were stored at -80°C on ice or at 4°C to thaw slowly. Repeated freezing and thawing cycles were avoided. According to the pre-experiment or the instructions, some samples may need to be appropriately diluted with the diluent specified in the kit before testing. After adding the substrate (TMB), colour development should be closely monitored or timed precisely. The colour development reaction usually occurs at room temperature in the dark. After the stop solution was added, the plate reading was completed within the specified time (usually within 30 minutes). The absorbance (OD) of the solution at 450 nm after the reaction was terminated was measured using an enzyme-linked immunosorbent assay (ELISA) reader. If required by the kit, 540 nm or 570 nm was used as the reference wavelength minus the background.

### Detection of serum SIRT1, ESM-1 and FGF21

Five millilitres of venous blood were drawn from each patient at admission. The blood was centrifuged at 3,000 r/min for 10 minutes with a centrifugation radius of 13.5 cm. For later use, the serum was extracted and stored at -80°C. A Multiskan FC fully automatic microplate reader (Thermo Fisher Scientific, USA) was used to measure serum levels of SIRT1, ESM-1, and FGF21 using an enzyme-linked immunosorbent assay.

Every operation stage was completed in compliance with the guidelines. Serum SIRT1 was measured using the Cloud-Clone SEA896Hu kit (USA, item #SEA896Hu), ESM-1 using the RayBiotech ELH-Endocan kit (USA, item #ELH-Endocan), and FGF21 using the R&D Systems DF2100 kit (USA, item #DF2100). Serum samples were stored in Eppendorf LoBind Tubes™ (Germany, item #022431081).

### Observation indicators

(1) There were two groups of baseline data. (2) Serum SIRT1, ESM-1 and FGF21 levels in the two groups. (3) Serum SIRT1, ESM-1 and FGF21 levels in patients with different disease severities. (4) Relationships between the levels of serum SIRT1, ESM-1 and FGF21 and the severity of the disease. (5) Correlations between serum SIRT1, ESM-1, and FGF21 levels and treatment outcomes. (6) The predictive value of serum SIRT1, ESM-1 and FGF21 for treatment outcomes.

### Statistical methods

The data were processed using SPSS 27.0. The measurement data are presented as (x̄±s). Further comparisons between paired groups were conducted via the least significant difference (LSD) test. Count data are expressed as [n(%)], and the χ^2^ test was used for comparison. The RIDIT test was used to compare the grade data, which are reported as [n (%)]. The associations between serum SIRT1, ESM-1, and FGF21 levels and illness severity were examined via the Spearman correlation coefficient. The associations between serum SIRT1, ESM-1, and FGF21 levels and treatment outcomes were investigated via partial correlation analysis. The predictive value of serum SIRT1, ESM-1 and FGF21 for treatment outcomes was analysed via receiver operating characteristic (ROC) curves. The area under the curve (AUC) was compared via the Z test.

## Results

### Comparative analysis of the baseline data between the two groups

There was no statistically significant difference in sex, age, basic medical history or infection site between the two groups (P>0.05). SIRT1 was consistently expressed at low levels in the death group, suggesting it may confer survival benefits as a protective factor. Both FGF21 and ESM-1 showed a significant upward trend in the death group. The increased expression of these genes might be related to aggravated pathological damage or compensatory dysregulation, especially the increase in FGF21. There are systematic differences in the clinical indicators reflecting disease severity and in the expression profiles of target markers between the survival and nonsurvival groups. The inhibitory expression of SIRT1 and the activating expression of FGF21 and ESM-1 constitute a biomarker combination that is significantly associated with poor prognosis, suggesting a synergistic pathological role in the course of ARDS.

There were statistically significant differences in disease severity and the length of time each group spent in the intensive care unit and on mechanical ventilation (P<0.05; see Table 1).

**Table 1:** Comparison of baseline data between groups [(x±)[n(%)].

Baseline data	Classification	Poor outcome group<br>(n=44)	Good outcome<br>group (n = 96)	t/x^2^	P
Gender	Male	25 (56.82)	57 (59.38)	0.081	0.776
	Female	19 (43.18)	39 (40.62)		
Age/Year		54.28±4.02	53.96±3.85	0.450	0.653
Basic medical history	Diabetes	6 (13.64)	11 (11.46)	0.134	0.714
	Hypertension	13 (29.54)	25 (26.04)	0.187	0.665
	Hyperlipidemia	8 (18.18)	15 (15.62)	0.144	0.705
Infection site	Pulmonary infection	20 (45.45)	45 (46.88)	0.105	0.916
	Abdominal cavity infection	10 (22.73)	20 (20.83)		
	Urinary tract infection	9 (20.45)	21 (21.88)		
	Others	5 (11.36)	10 (10.42)		
Severity of the illness	Mild	8 (18.18)	37 (38.54)	2.626	0.009
	Moderate	18 (40.91)	38 (39.58)		
	Severe	18 (40.91)	21 (21.88)		
Mechanical ventilation<br>time/day		4.68±1.03	3.39±0.86	7.733	<0.001
Length of stay in the<br>ICU per day		10.21±2.15	7.44±1.27	9.527	<0.001

### Comparison of serum SIRT1, ESM-1 and FGF21 levels between the two groups

While ESM-1 and FGF21 levels were higher in the poor outcome group than in the good outcome group, serum SIRT1 levels were lower in the poor outcome group. Table 2 shows that the differences were statistically significant (P<0.05).

**Table 2:** Serum SIRT1, ESM-1, and FGF21 levels between groups.

Group	n	SIRT1/<br>(ng/mL)	ESM-1/<br>(ng/mL)	FGF21/<br>(pg/mL)
Poor outcome<br>group	44	0.33±0.09	3.87±1.10	803.52±246.35
Good outcome<br>group	96	0.48±0.15	2.94±0.85	557.91±174.44

Compared with those in the adverse outcome group, baseline serum SIRT1 levels in the good outcome group were significantly higher, whereas ESM-1 and FGF21 levels were substantially lower (all P<0.05). With improvements in therapeutic effect, SIRT1 expression tended to increase, whereas ESM-1 and FGF21 expression tended to decrease. Overall, SIRT1 is positively correlated with treatment outcome, whereas ESM-1 and FGF21 are associated with adverse outcomes, suggesting that endothelial function and the metabolic stress state are closely linked to the therapeutic effect in ARDS.

### Comparison of serum SIRT1, ESM-1 and FGF21 levels in patients with different disease severities

One-way analysis of variance revealed that serum SIRT1 levels decreased gradually across mild, moderate, and severe patients, whereas ESM-1 and FGF21 levels increased gradually across mild, moderate, and severe patients. Table 3 indicates that the difference was statistically significant (P<0.05). The level of SIRT1 decreased gradually from light to heavy, whereas the levels of ESM-1 and FGF21 increased gradually (the differences between groups were all P<0.05, and the trend test was P<0.05). Pairwise comparisons mostly revealed significant differences. Correlation analysis showed that SIRT1 was positively correlated with PaO_2_/FiO_2_ and negatively correlated with APACHE II and SOFA scores, whereas ESM-1 and FGF21 were negatively correlated with PaO_2_/FiO_2_ and positively correlated with severity scores.

**Table 3:** Serum SIRT1, ESM-1 and FGF21 levels in patients with different disease severity.

Group	n	SIRT1/<br>(ng/mL)	ESM-1/<br>(ng/mL)	FGF21/<br>(pg/mL)
Mild<br>group	45	0.54±0.12	2.35±0.57	528.49±102.64
Moderate<br>group	56	0.43±0.11	3.17±0.63	636.07±118.75
Severe<br>group	39	0.30±0.08	4.33±0.80	756.72±141.53

### Relationships between serum SIRT1, ESM-1, and FGF21 levels and disease severity

**Figure 1:**
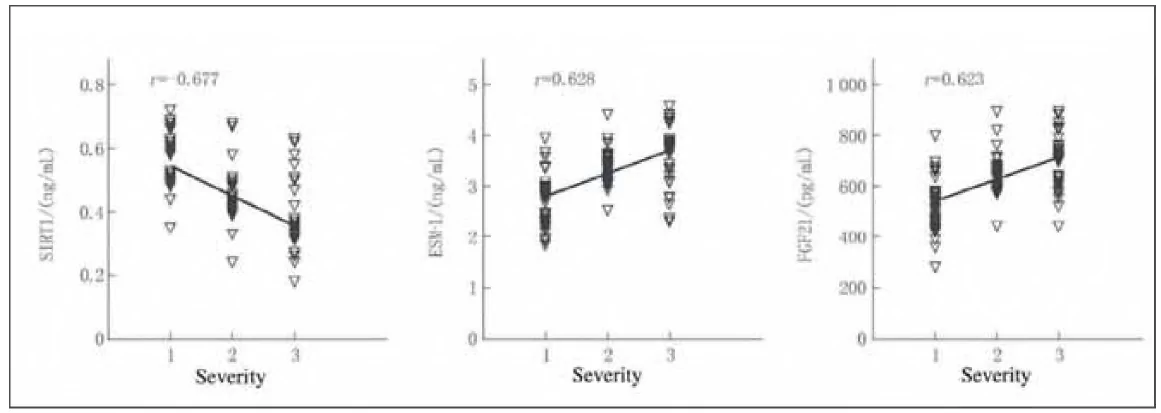
Changes in Phlebitis Grading and VAS Scores; (A) Phlebitis Grading; (B) VAS Scores.

According to the Spearman correlation analysis shown in Figure 1, serum SIRT1 was negatively associated with illness severity (mild =1, moderate =2, severe =3) (P<0.05), whereas ESM-1 and FGF21 were positively correlated (mild =1, moderate =2, severe =3) (P<0.05). Correlation analysis revealed that serum SIRT1 was significantly negatively correlated with disease severity (negatively correlated with the APACHE II score and SOFA score and positively correlated with the PaO_2_/FiO_2_), whereas ESM-1 and FGF21 were positively correlated with disease severity (positively correlated with the APACHE II score and SOFA score) and negatively correlated with the PaO_2_/FiO_2_ ratio (all P<0.05). A trend test revealed that, with increasing disease severity, SIRT1 decreased gradually, whereas ESM-1 and FGF21 increased gradually. After adjusting for multiple factors, such as age, comorbidities, and infection-related indicators, the above associations remained independent.

### Correlations between serum SIRT1, ESM-1, and FGF21 levels and treatment outcomes

To examine correlations between serum SIRT1, ESM-1, and FGF21 levels in patients with sepsis-related ARDS, and to eliminate the interference of other statistically significant factors (mechanical ventilation time, ICU stay time, and disease severity), partial correlation analysis was performed after controlling for these factors. As indicated in Table 4, the findings demonstrated a substantial correlation (P<0.001) between the treatment outcomes of patients with sepsis-related ARDS and the serum levels of SIRT1, ESM-1, and FGF21.

**Table 4:** Correlation between serum SIRT1, ESM-1, FGF21 and treatment outcomes.

Variable	Partial correlation<br>coefficient	95%CI	P
SIRT1	-0.790	0.685 0.911	<0.001
ESM-1	0.811	0.702 0.938	<0.001
FGF21	0.749	0.620 0.905	<0.001

### Predictive value of serum SIRT1, ESM-1, and FGF21

Serum SIRT1, ESM-1, and FGF21 levels in the two groups were used as source data to calculate the treatment outcomes. The ROC curve was then plotted, with the good-outcome group as the negative class and the poor-outcome group as the positive class. The AUCs for serum SIRT1, ESM-1, and FGF21 in predicting the treatment outcome of patients with sepsis-related ARDS were 0.742, 0.838, and 0.796, respectively; the sensitivities were 77.27%, 77.27%, and 70.45%, respectively; and the specificities were 64.58%, 81.25%, and 87.50%, respectively. When a patient was treated for acute respiratory distress syndrome associated with sepsis, the total AUC of the three markers was 0.939, with a sensitivity of 88.64% and a specificity of 83.33%, which was significantly greater than the individual predictive value of the three indicators alone (P< 0.05), as shown in Table 5.

**Table 5:** Comparison of the efficacy of joint prediction and individual prediction of each indicator.

Pairwise<br>comparison	AUC<br>difference	Standard<br>error	95%CI	Z	P
United:<br>SIRT1	0.197	0.046	0.107<br>0.287	4.283	<0.001
United:<br>ESM-1	0.101	0.045	0.012<br>0.190	2.235	0.025
United:<br>FGF21	0.143	0.050	0.046<br>0.240	2.887	0.004

## Discussion

SIRT1 is a histone deacetylase that is widely expressed in the heart, kidneys, liver, pancreas, and bones [17]. It has anti-inflammatory and antioxidant stress effects and participates in the processes of cardiovascular and cerebrovascular diseases and inflammatory damage. SIRT1 levels are low in sepsis patients and even lower in those with ARDS. Serum SIRT1 is negatively correlated with the severity of sepsis-related ARDS [18][19][20]. These findings suggest that SIRT1 can exert a strong anti-inflammatory effect through the NF-kB signalling pathway and regulate oxidative stress-related signalling pathways to achieve antioxidative stress effects. It can inhibit the inflammatory response in pulmonary vessels, oxidative stress, and apoptosis of alveolar epithelial cells, and delay the progression of the disease. Another study [21] confirmed that SIRT1 was significantly expressed at low levels in a mouse model of acute lung injury and could inhibit endotoxin-induced lung tissue damage. Therefore, SIRT1 is significantly associated not only with the severity of sepsis-related ARDS but also with treatment outcomes [22]. The partial correlation results of this study have also been confirmed. ROC curve analysis revealed that serum SIRT1 concentration could predict the treatment outcome in sepsis-related ARDS patients, providing a relevant reference for clinical practice.

ESM-1 is a soluble dermatin sulfate proteoglycan that can bind to various adhesion molecules and participate in cell growth, proliferation, apoptosis and various cell signal transduction processes. It is released in large quantities when endothelial cells are abnormally activated or have functional disorders. Relevant studies [23][24][25] have shown that increased permeability of the alveolar-capillary membrane and damage to the alveolar capillary barrier are among the main mechanisms causing sepsis-related ARDS [26]. In the context of sepsis-induced inflammation, endothelial cells are activated and exhibit a range of phenotypic changes. During this process, excessive amounts of ESM-1 are synthesised and secreted, intensifying the inflammatory response, disrupting the alveolar capillary barrier, increasing alveolar capillary membrane permeability, and accelerating the onset and progression of sepsis-related ARD [27][28][29]. Therefore, serum ESM-1 can be closely associated with the severity and treatment outcome of sepsis-related ARDS via the abovementioned mechanism of action. This study also revealed that the serum ESM-1 level can independently predict the treatment outcome of sepsis-related ARDS patients and can be used as an independent predictor to assist in the early clinical prediction of treatment outcomes [30].

FGF21 can regulate metabolic homeostasis throughout the body and modulate the inflammatory response [31]. This study revealed that serum FGF21 was positively correlated with the severity of sepsis-related ARDS, consistent with results from another study. FGF21 can inhibit pulmonary cell apoptosis and inflammatory responses by regulating related signalling pathways, thereby affecting the development of ARDS. Moreover, under the infection-stress state induced by sepsis, many inflammatory factors are excessively released, leading to excessive activation of the NF-kB signalling pathway, which, in turn, causes abnormal accumulation of FGF21 in peripheral blood. This further leads to a significant increase in their serum levels during sepsis development. Partial correlation analysis revealed that serum FGF21 was significantly correlated with treatment outcome in patients with sepsis-related ARDS, suggesting that it can serve as a significant predictor of treatment outcome [32]. The AUC of serum FGF21 for predicting treatment outcome in patients with sepsis-related ARDS is close to 0.8, indicating good predictive value. The practical value of a single indicator for evaluating a condition or predicting prognosis in clinical practice is limited. This study analysed the value of combining serum SIRT1, ESM-1, and FGF21 predictions in predicting treatment outcomes in patients with sepsis-related ARDS. The combined predictive value was more reliable and could provide more accurate and effective data support for clinical practice [33]. Owing to limitations in clinical conditions and time, the combined prediction method for serum SIRT1, ESM-1, and FGF21 has not yet been applied in clinical practice and awaits further verification.

In conclusion, serum SIRT1, ESM-1, and FGF21 levels in individuals with sepsis-related ARDS are highly correlated with illness severity and can be used to gauge disease severity. Moreover, they are related to treatment outcomes and can independently predict them, with a higher combined predictive value.

## Dodatak

### Conflict of interest statement

All the authors declare that they have no conflict of interest in this work.
